# Oral nutrition for left‐sided malignant colonic obstruction after decompression with a transanal drainage tube: A case series of 6 patients

**DOI:** 10.1002/ccr3.1616

**Published:** 2018-05-29

**Authors:** Shigehiro Kojima, Tsuguo Sakamoto, Yuko Nagai, Kazuhito Yajima

**Affiliations:** ^1^ Department of Surgery Sainokuni Higashiomiya Medical Center Saitama Japan

**Keywords:** bridge to surgery, colorectal cancer, malignant bowel obstruction, obstructive colon cancer, self‐expandable metal stent

## Abstract

Oral nutrition with a low‐residue diet for left‐sided malignant colonic obstruction after decompression with a transanal drainage tube is safe and can be considered a viable preoperative management option for appropriate patients.

## 1 INTRODUCTION

The best preoperative management for acute malignant left‐sided large bowel obstruction is still under debate. Oral nutrition with a low‐residue diet for left‐sided malignant colonic obstruction after decompression with a transanal drainage tube is safe and can be considered a viable preoperative management option for appropriate patients.

Malignant colonic obstruction occurs in 8%‐13% of patients with colonic cancer.^1^, ^2^, ^3^ Surgical emergencies are associated with high morbidity and mortality rates and poor long‐term survival.^4^, ^5^, ^6^ The best surgical treatment for acute malignant left‐sided large bowel obstruction remains controversial. However, elective one‐stage resection and anastomosis after decompression with a transanal drainage tube (TDT) or a self‐expandable metal stent (SEMS) are expected to improve the postoperative outcome.^7^, ^8^, ^9^, ^10^, ^11^ The mortality rate for patients with colorectal cancer undergoing emergency surgery reportedly ranges from 8% to 15%, compared with 3% to 6% mortality in those undergoing elective surgery.^4^, ^6^, ^12^

The superiority of outcomes after elective one‐stage operations compared with emergency surgery can be attributed to several factors. One key aspect is the time interval, which allows for improvement of the patient’s general condition.^13^, ^14^ Patients with obstructive colorectal cancer often have varying degrees of chronic malnutrition, and preoperative nutritional management is mandatory to improve postoperative outcomes. However, few reports have addressed preoperative nutritional management after TDT placement.

We primarily use TDT placement as a bridge to elective surgery for acute left‐sided malignant colorectal obstruction. Since 2015, we have provided oral nutrition with a low‐residue diet (LRD) after decompression with a TDT. We herein report this treatment approach and evaluate its clinical safety.

## 2 CASE PRESENTATION

### 2.1 Patients

In our department, TDT placement as a bridge to elective surgery is performed for patients with left‐sided complete bowel obstruction, except those with suspected or impending perforation or direct invasion to other organs; a SEMS is used for palliation, with no adjunctive chemotherapy or surgery. From January 2015 to September 2017, 61 patients with left‐sided colorectal cancer were treated by the first author at Sainokuni Higashiomiya Medical Center. Of the 61 patients, 8 had complete bowel obstruction. Of these 8 patients, 2 were excluded from this study because they needed emergency surgery (one for perforation and invasion to the small intestine, and one for impending perforation). The remaining 6 patients were treated with oral nutrition after decompression with a TDT. The median age of the patients was 67 years (range, 54‐80 years). The primary tumor location was the sigmoid colon in 3 patients and the descending colon in 3 patients. Two patients were diagnosed with bacteremia due to obstructive colitis based on the presence of a high fever and blood culture results. Two were diagnosed with clinical stage IV cancer following preoperative examinations. The patients’ characteristics are summarized in Table 1.

**Table 1:** Patients’ characteristics

Case	Age (y)	Sex	PS	Comorbidity	ASA grade	Bacteremia due to obstructive colitis	Tumor location	UICC stage
1	65	M	0	None	II	Yes	Descending	IV
2	54	M	1	None	I	No	Descending	IIIB
3	80	M	1	HT	II	No	Sigmoid	IIIB
4	68	F	0	None	II	No	Sigmoid	IIB
5	66	F	1	DM, HT	III	Yes	Descending	IV
6	71	F	1	DM	II	No	Sigmoid	IIIC

ASA, American Society of Anesthesiologists; BMI, body mass index; DM, diabetes mellitus; HT, hypertension; PS, performance status; UICC, International Union Against Cancer.

### 2.2 Preoperative management

The detailed methods of TDT placement have been previously described.^7^, ^8^, ^9^, ^10^ The TDT used in our department was constructed with silicone, had an outer diameter of 7.3 mm (22 Fr) and length of 120 cm, and had an open tip with 4 side holes (Transanal Ileus Tube Set; Create Medic Co., Yokohama, Japan). TDT placements were performed under fluoroscopic and endoscopic guidance. After the obstructive site was identified using an endoscope, endoscopic biopsy was performed for histological diagnosis. A guidewire was introduced through the endoscope and through the tumor beyond the point of obstruction. The endoscope was then removed, and a TDT was introduced over the remaining guidewire. After confirming fluoroscopically that the balloon part of the tube had passed the obstructive lesion, the balloon was inflated with 20 mL of distilled water to prevent tube migration. A negative‐pressure aspirator was used for decompression. The intestinal tract was cleaned once a day using 200‐400 mL of water, and tube flushing was performed 3 times a day using 30 mL of water until the operation. Only peripheral parenteral nutrition (PPN) was undertaken in all patients before starting oral nutrition.

One week after TDT placement, an abdominal computed tomography (CT) examination was performed to observe the tube position and bowel decompression. CT scans were repeatedly performed at 1‐week intervals to monitor for possible insufficient decompression. After confirmation of sufficient decompression with a TDT, preoperative oral nutrition using an immune‐enhancing LRD (MEIN^®^; Meiji Dairies, Tokyo, Japan) was initiated. Minimum calorie administration of the LRD was 600 kcal/d, and the target total calorie administration in combination with PPN was set at a minimum of 20 kcal/kg/d. Sodium picosulfate was administered, and a negative‐pressure aspirator was used continuously during oral nutrition with the LRD. An abdominal radiograph was obtained every 2‐3 days to confirm the bowel decompression and tube position. A previous randomized controlled trial showed that a short interval of 5‐14 days after SEMS insertion was associated with a high rate of leakage.^15^ Therefore, after 1‐3 weeks of oral nutrition (ie, 2‐4 weeks after TDT placement), we performed laparoscopic one‐stage resection and anastomosis. The TDT was removed before commencing the operation.

### 2.3 Perioperative surgical data

The technical success rate of TDT placements was 100% (6/6). A long time (approximately 12 days) was required to obtain sufficient bowel decompression in Patient 4, and the interval before starting oral nutrition was extended to 2 weeks. In the remaining 5 patients, oral nutrition was started 1 week after TDT placement.

Oral nutrition continued normally in all patients without bowel reobstruction, and no worsening of abdominal symptoms occurred. The preoperative period was extended in 2 patients to treat bacteremia resulting from obstructive colitis (Patient 5) and peripheral venous catheter‐associated phlebitis (Patient 6). The perioperative management conditions are shown in Table 2.

**Table 2:** Preoperative management and perioperative data

Case	Successful placement of TDT	Interval before surgery after TDT placement (d)	Maximum enteral nutrition (kcal)	Total calorie administration (kcal/kg)	Admission	BMI (kg/m^2^) operation	Discharge
1	Yes	17	600	22.2	15.6	14.5	14.5
2	Yes	14	600	19.4	19.1	18.6	18.6
3	Yes	18	800	27.6	21.5	20.1	19.5
4	Yes	24	1000	25.9	26.5	24.4	23.2
5	Yes	28	900	23.1	27.0	24.8	24.2
6	Yes	23	600	26.9	22.8	20.6	20.3

BMI, body mass index; TDT; transanal drainage tube.

No bowel distension (a potential hindrance to surgery) occurred intraoperatively, and all patients underwent a successful laparoscopic procedure without conversion to open surgery. Postoperative anastomotic leakage occurred in 1 patient and was cured by nonsurgical treatment. The median postoperative hospital stay was 10 days (range, 8‐34 days). During the median postoperative follow‐up of 11 months (range, 1‐30 months), no recurrence occurred in 4 patients diagnosed with stage II or III cancer, while the 2 patients with stage IV cancer continued to receive systemic chemotherapy. The perioperative surgical data of the patients are summarized in Table 3.

**Table 3:** Surgical data

Case	Surgical procedure (Anastomosis)	Colostomy formation	Operative duration (min)	Blood loss (mL)	Postoperative bowel obstruction	AL	Postoperative hospital stay (d)
1	LAP (FEEA)	No	220	3	No	No	8
2	LAP (FEEA)	No	253	20	No	No	11
3	LAP (DST)	No	222	10	No	No	9
4	LAP (DST)	No	217	5	No	Yes	34
5	LAP (FEEA)	No	298	100	No	No	10
6	LAP (DST)	No	193	2	No	No	10

AL, anastomotic leakage; DST, double stapling technique; FEEA, functional end‐to‐end anastomosis; LAP, laparoscopic colectomy.

### 2.4 Perioperative nutritional data

**Figure 1:**
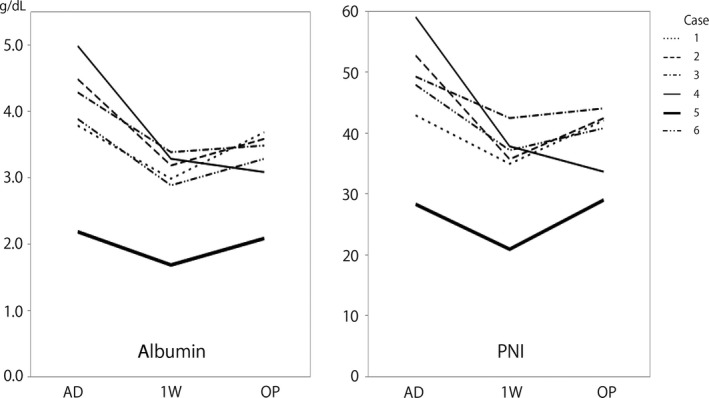
Line graph showing the time‐series changes in the serum albumin concentration and PNI. Five of six patients showed an increasing trend after correction of dehydration. In Patient 4, who developed postoperative anastomotic leakage, the PNI continued to decrease until the operation. AD, admission; 1 W, 1 week after placement of a transanal drainage tube; OP, operation; PNI, prognostic nutritional index

**Figure 2:**
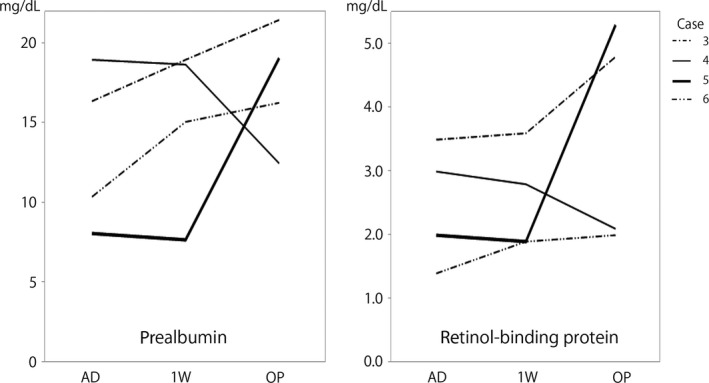
Line graph showing the time‐series changes in the serum concentration of rapid turnover proteins. The serum concentrations of rapid turnover proteins continued to worsen until the operation in Patient 4. AD, admission; 1 W, 1 week after placement of a transanal drainage tube; OP, operation

Each patient’s nutritional condition was evaluated using Onodera’s prognostic nutritional index (PNI), calculated as follows: [10× serum albumin value (g/dL)] + [0.005 × peripheral lymphocyte count (count/mm^3^)]. The PNI was designed to assess the nutritional status of patients undergoing surgery for gastrointestinal malignancy.^16^ Although the basis of the independent correlation between the PNI and postoperative outcome is not clear, the preoperative PNI is recognized as a useful predictor of postoperative complications and survival in patients with colorectal cancer.^17^ In all patients, the PNI decreased due to a decrease in the serum albumin concentration (reflecting correction of dehydration) 1 week after TDT placement; however, the PNI increased again until the time of surgery in 5 of the 6 patients. In 2 patients, the PNI was <40 before surgery (Figure 1). In addition to the PNI, the serum concentrations of rapid turnover proteins, such as prealbumin and retinol‐binding protein, were examined in Patients 3 to 6 (Figure 2). In the patient with postoperative anastomotic leakage, all evaluation items continued to decrease until the time of surgery.

## 3 DISCUSSION

The present results indicate the safety of preoperative management by oral nutrition with an LRD after decompression with a TDT for patients with malignant left‐sided colonic obstruction. Oral nutrition with an LRD was well tolerated by our cohort of 6 patients. In all 6 patients, abdominal symptoms did not worsen after initiation of the LRD. All patients successfully underwent a one‐stage laparoscopic operation without conversion to open surgery. A good outcome with regard to the mortality rate (0%) was observed compared with previous reports.^4^, ^6^, ^12^ Therefore, we believe that oral nutrition with an LRD for left‐sided malignant colonic obstruction after decompression with a TDT is safe and can be considered a viable option for the practical preoperative management of affected patients.

In a previous retrospective cohort study, decompression with a TDT was superior to urgent surgery and insertion of a SEMS because of its high rates of clinical success and one‐stage laparoscopic surgery. The study indicated that decompression with a TDT represents an attractive option for the management of obstructive colon cancer.^7^ However, a TDT has several disadvantages mainly attributable to its small outer diameter compared with a SEMS. To avoid the risk of bowel reobstruction, total parenteral nutrition (TPN) is most frequently used to administer preoperative nutrition in patients undergoing drainage by a TDT. In the present series, TPN was replaced by a combination of oral nutrition and PPN. Central venous catheterization should be avoided if possible, especially because of the potential risk of bacterial translocation due to obstructive colitis.

The use of strict patient selection criteria and attentive observation are essential in the preoperative management of patients with obstructive colon cancer using a TDT and oral nutrition. In previous studies, decompression with a TDT was complicated by perforation in 0%‐7% of patients.^7^, ^9^ To avoid the risk of bowel perforation caused by a TDT in the present study, we excluded patients with right colonic obstruction, suspected or impending perforation, or direct cancer invasion to other organs. After TDT placement, an abdominal radiographic or CT examination was performed as necessary to observe the tube position and bowel decompression. An abdominal CT examination was performed at least once after TDT placement in the present study because the extent to which oral nutrition with the LRD affected the obstructed colon was unknown. Additionally, we consider that CT is superior to radiography for the appropriate assessment of bowel decompression and for identification of silent perforation until sufficient decompression is confirmed. No bowel perforation or reobstruction was observed in our case series. From the viewpoint of radiation exposure, radiography alone may be adequate for assessment of the bowel condition if the patient’s abdominal symptoms improve with sufficient drainage by the TDT after placement.

There is no disagreement regarding avoidance of long periods of preoperative fasting.^18^ Combined nutritional therapy consisting of PPN and a small amount of an LRD can prevent decreases in intestinal mucosal integrity and provide better nutritional support than TPN for surgical patients.^19^ In our cases, nutritional evaluation showed an increase in the PNI after correction of dehydration in 5 of the 6 patients. The remaining patient exhibited worsening of all nutritional indicators. It took a long time for this patient to obtain sufficient bowel decompression after TDT placement, and she also had postoperative anastomotic leakage. The reason for the unfavorable preoperative course in this patient was not clear. However, during such a preoperative course or when the PNI is <40, TPN should be considered and one‐stage resection and anastomosis should probably be avoided or a diverting ileostomy should be created.

There is significant concern regarding the cost of preoperative management with a TDT, especially compared with a SEMS. A simple comparison of cost for a one‐stage operation with a TDT versus a bridge to surgery with a SEMS is impossible because preoperative management with a TDT requires admission for several weeks before surgery. However, several studies have shown that the cost of a TDT ($500) is much lower than the cost of a SEMS ($2000).^9^, ^10^, ^20^ Furthermore, in patients undergoing management with a TDT, combined nutritional therapy involving an LRD and PPN can reduce the cost of nutritional management compared with TPN.

It is difficult to determine the nutritional and surgical benefits of our preoperative management in comparison with emergency surgery or a bridge to elective surgery with a SEMS using the results of our small case series. As a bridge to elective surgery, a SEMS has several advantages over a TDT: it does not necessarily require admission before surgery, no irrigation is needed, the patient experiences no discomfort, it produces no foul smell, and it allows for earlier oral intake. However, the mortality and morbidity rates in emergency surgery are higher than those in elective surgery for patients with acute colorectal obstruction,^4^, ^6^, ^12^ and some studies have revealed the oncological risks of using a SEMS.^21^, ^22^ TDT placement is the first choice for left‐sided malignant colonic obstruction in our department because postoperative long‐term survival is thought to be one of the most essentially important outcomes, and the long‐term oncological outcome of SEMS placement is controversial. The European Society of Gastrointestinal Endoscopy does not recommend the use of a SEMS as a bridge to elective surgery for standard treatment of potentially curable patients with left‐sided malignant colonic obstruction.^23^ Overall, elective one‐stage resection and anastomosis for stage IV cancer are controversial from the viewpoint of future chemotherapy; however, we believe that decompression with a TDT is a valuable option for the management of malignant left‐sided colorectal obstruction. Further research is needed to determine the nutritional and surgical advantages of our preoperative management, evaluate the nutritional regimen, and elucidate the details of the strategy, such as determining the nutritional goal of LRD administration and the optimal dose of oral nutritional agents.

In conclusion, oral nutrition with an LRD for left‐sided malignant colonic obstruction after decompression with a TDT is safe and can be considered a viable and practical preoperative management approach for patients without perforation or direct invasion to adjacent organs.

## CONSENT FOR PUBLICATION

Written informed consent was obtained from the patients for publication of this article.

## CONFLICT OF INTERESTS

The authors declare that they have no competing interests.

## AUTHORS’ CONTRIBUTIONS

SK: conceptualized the study, analyzed the data, and drafted the manuscript. YN: assisted with conceptualization of the study. TS and KY: contributed to the drafting and revision of the manuscript.
